# Characterization of the intrahippocampal kainic acid model in female mice with a special focus on seizure suppression by antiseizure medications

**DOI:** 10.1016/j.expneurol.2024.114749

**Published:** 2024-03-09

**Authors:** Melanie Widmann, Andreas Lieb, Barbara Fogli, Angela Steck, Anna Mutti, Christoph Schwarzer

**Affiliations:** Department of Pharmacology, Medical University of Innsbruck, Innsbruck, Austria

**Keywords:** Temporal lobe epilepsy, Hippocampal paroxysmal discharges, Drug-resistant seizures, Lamotrigine, Oxcarbazepine, Levetiracetam

## Abstract

Despite special challenges in the medical treatment of women with epilepsy, in particular preclinical animal studies were focused on males for decades and females have only recently moved into the focus of scientific interest. The intrahippocampal kainic acid (IHKA) mouse model of temporal lobe epilepsy (TLE) is one of the most studied models in males reproducing electroencephalographic (EEG) and histopathological features of human TLE. Hippocampal paroxysmal discharges (HPDs) were described as drug resistant focal seizures in males. Here, we investigated the IHKA model in female mice, in particular drug-resistance of HPDs and the influence of antiseizure medications (ASMs) on the power spectrum.

After injecting kainic acid (KA) unilaterally into the hippocampus of female mice, we monitored the development of epileptiform activity by local field potential (LFP) recordings. Subsequently, we evaluated the effect of the commonly prescribed ASMs lamotrigine (LTG), oxcarbazepine (OXC) and levetiracetam (LEV), as well as the benzodiazepine diazepam (DZP) with a focus on HPDs and power spectral analysis and assessed neuropathological alterations of the hippocampus.

In the IHKA model, female mice replicated key features of human TLE as previously described in males. Importantly, HPDs in female mice did not respond to commonly prescribed ASMs in line with the drug-resistance in males, thus representing a suitable model of drug-resistant seizures. Intriguingly, we observed an increased occurrence of generalized seizures after LTG. Power spectral analysis revealed a pronounced increase in the delta frequency range after the higher dose of 30 mg/kg LTG. DZP abolished HPDs and caused a marked reduction over a wide frequency range (delta, theta, and alpha) of the power spectrum.

By characterizing the IHKA model of TLE in female mice we address an important gap in basic research. Considering the special challenges complicating the therapeutic management of epilepsy in women, inclusion of females in preclinical studies is imperative. A well-characterized female model is a prerequisite for the development of novel therapeutic strategies tailored to sex-specific needs and for studies on the effect of epilepsy and ASMs during pregnancy.

## 1 Introduction

Affecting around 50 million people of both sexes and all ages, epilepsies are among the most prevalent neurological diseases worldwide (Fiest et al., 2017) and contribute significantly to the global burden of diseases with an estimated proportion of > 0.5% (Beghi et al., 2019). Although males and females display a similar prevalence, the medical management of women with epilepsy is particularly challenging due to complex interactions between sex hormones, seizures and antiseizure medications (ASMs) as well as special needs during pregnancy (for review see (Harden and Pennell, 2013; Stephen et al., 2019)). For instance, it has been shown that lamotrigine (LTG) serum levels vary with menstrual cycle phase and oral contraceptive use, potentially leading to loss of seizure control (Herzog et al., 2009). While women with epilepsy face special challenges, previous research, in particular preclinical animal studies primarily focused on males and females have only recently moved into the focus of scientific interest. However, a well-characterized female epilepsy model would be a prerequisite for the development of novel therapeutic strategies tailored to gender-specific needs and for studies on the effect of epilepsy and ASMs during pregnancy.

One of the most frequently studied models of temporal lobe epilepsy (TLE) is the intrahippocampal kainic acid (IHKA) mouse model, which reproduces several EEG and histopathological features of human TLE (Bouilleret et al., 2000; Riban et al., 2002; Suzuki et al., 1995). Hippocampal paroxysmal discharges (HPDs) were described as drug resistant focal seizures in males (Duveau et al., 2016; Riban et al., 2002; Zangrandi et al., 2016) and have a high predictive value for the screening of novel drugs (Kehne et al., 2017). However, there is still a remarkable lack of data on females, particularly drug-responsiveness of HPDs has not been investigated yet.

Therefore, the objective of this study was to further characterize the IHKA mouse model of TLE in female mice, in particular the effect of ASMs on HPDs and on the power spectrum.

## 2 Materials and methods

### 2.1 Intrahippocampal kainic acid injection and electrode implantation

Stereotaxic surgery with IHKA injection and implantation of local field potential (LFP) electrodes was performed as previously described (Zangrandi et al., 2016). Briefly, a bolus of 50 nl 20 mM kainic acid solution (Ocean Produce International, prepared in purified water) was injected into the CA1 area of the left hippocampus (AP -1.8 mm, ML +1.2 mm, DV -1.8 mm in reference to bregma) via a 30 G cannula attached to a 0.5 μl Hamilton syringe. After 10 min waiting time, the cannula was gradually withdrawn to minimize backflow along the injection path.

**Fig. 1:**
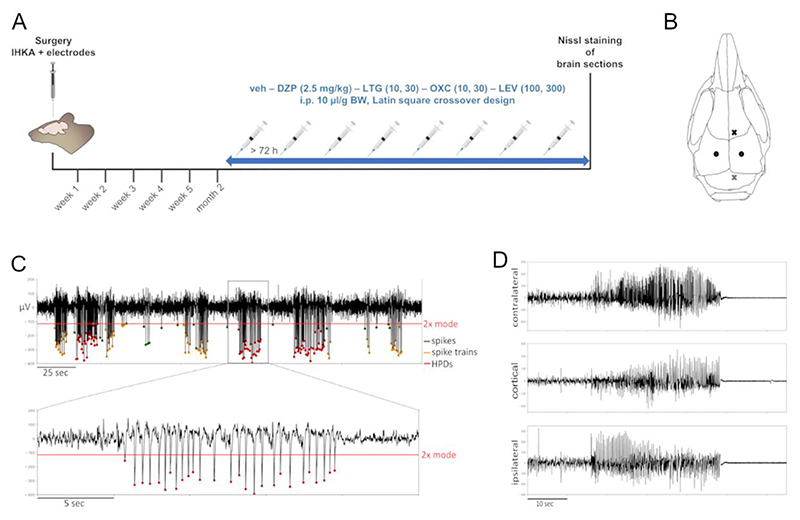
Experimental timeline, schematic of the electrode implantation sites and representative LFP traces. (A) Experimental timeline for monitoring the development of epileptiform activity following IHKA in female mice. Females with established chronic epilepsy (>50 s/h ipsilateral HPDs) were subsequently tested with DZP (2.5 mg/kg), LTG (10 and 30 mg/kg), OXC (10 and 30 mg/kg) and LEV (100 and 300 mg/kg) compared to vehicle (10% DMSO and 90% saline with 3% Tween 80) i.p. in a volume of 10 μl/g. Treatment schedules were designed according to a Latin square crossover design with 72 h minimum resting period between injections. Afterwards, Nissl-stained brain sections were analyzed for morphological alterations. IHKA, intrahippocampal kainic acid; veh, vehicle; DZP, diazepam; LTG, lamotrigine; OXC, oxcarbazepine; LEV, levetiracetam; i.p., intraperitoneal; BW, body weight. (B) Schematic figure showing the electrode implantation sites. The black dots symbolize the 2 depth electrodes targeting the ipsilateral and the contralateral hippocampus (AP -1.8, ML ±1.3, DV -1.8). The black cross represents the single surface electrode implanted above the motor cortex, the grey cross the triple electrode for grounding and references positioned above the cerebellum. (C) Representative LFP trace of the ipsilateral hippocampus showing single spikes, spike trains and HPDs. Epileptiform spikes are characterized by an amplitude 2× mode (green dots represent single spikes). Spike trains (marked with orange dots) are series of at least 3 epileptiform spikes lasting 1–10 s with ≥1.33 Hz. Trains of epileptiform spikes ≥10 s are counted as HPDs (red dots). In this exemplary trace the spike polarity is negative, a minority of animals, however, present with positive spikes. Red line = baseline for spike threshold detection set to amplitude minimum of 2× mode. (D) LFP traces of a typical generalized seizure with approximately 30 s of high-amplitude, high-frequency discharges simultaneously both in the ipsi- and contralateral hippocampus as well as in the cortical electrode followed by a pronounced post-ictal depression of LFP activity.

Following KA injection, 2 depth electrodes consisting of a tungsten wire coated with epoxylite (diameter uncoated = 250 μm, FHC Inc., USA) soldered to a Teflon®-PFA-coated stainless steel wire (diameter uncoated = 203 μm, Science Products GmbH, Germany) ending with a gilded brass pin (Harwin, UK) were implanted bilaterally into CA1 (AP -1.8, ML ±1.3, DV -1.8) (Fig. 1 B). Screw electrodes were secured above the motor cortex to record surface LFPs and over the cerebellum as grounding and reference. All electrodes were stabilized to the skull surface with Paladur® dental cement (Kulzer GmbH).

### 2.2 Animals

All experimental procedures involving animals were designed in compliance with the principles outlined in the ARRIVE guidelines and the Basel declaration including the 3R (replacement, reduction, refinement) concept. All animal experiments were approved by the Austrian Animal Testing Commission (Austrian Federal Ministry of Education, Science and Research) in accordance with the EU Directive 2010/63/EU.

Female adult C57BL/6N mice (8–10 weeks at time of surgery) were used, either bred in-house or purchased from Charles River Germany. For breeding and maintenance, mice were group-housed (max. 5 mice in type II-L individually ventilated cages) under standard laboratory conditions with 12 h light-dark cycle (lights on 7 am - 7 pm) and freely accessible food and water. Post-surgery mice were single-caged.

### 2.3 LFP recordings and analyses and experimental treatments

To monitor the development of epileptiform activity, 18 female mice were recorded once per week for 24 h for the first 5 weeks and at 2 months after IHKA injection. Mice with established chronic epilepsy (meeting the inclusion criterium of >50 s/h HPDs at the ipsilateral recording electrode at 2 months, termed high-HPDs group, *n* = 7) were subjected to subsequent experimental treatments. 10 and 30 mg/kg LTG, 10 and 30 mg/kg OXC, 100 and 300 mg/kg LEV (all from BioTechne), 2.5 mg/kg DZP (Takeda Austria GmbH) and vehicle (10% DMSO and 90% saline with 3% Tween 80) were administered i.p. in a volume of 10 μl/g body weight according to a Latin square crossover design. The minimum resting period between injections was 72 h.

For LFP acquisition in freely moving animals, wireless devices (Neurologger from either TSE systems or Evolocus) simultaneously recording 4 channels (sampling rate 512 Hz or 500 Hz depending on manufacturer) were used. All recordings were performed in the home cages placed within a recording chamber covered by a mesh of conductive wire to block electromagnetic fields.

LFP traces were filtered between 0.5 and 70 Hz using a second-order Butterworth bandpass filter. Subsequently, the data were divided into 1 s long intervals of which the minimum or maximum (depending on epileptic spike polarity) was determined and the mode calculated. The baseline for spike threshold detection was set to an amplitude minimum or maximum of twice the mode calculated above. Spike trains were defined as series of at least 3 epileptiform spikes (amplitude 2× mode, minimum distance between spikes 70 ms) lasting 1–10 s with a minimum of 1.33 Hz. Trains of epileptiform spikes with a duration ≥10 s were counted as HPDs (Fig. 1 B). In addition, the LFP signals were visually scanned for generalized seizures (Fig. 1 C) using LabChart Reader version 8.1.14 (ADInstruments). Generalized seizures were defined as high-amplitude, high-frequency discharges simultaneously present in all channels followed by a clear post-ictal depression of LFP signals.

To evaluate the effect of treatments on epileptiform activity, the number and cumulative duration of spike trains and HPDs in the ipsilateral hippocampus were assessed between 35 and 94 min after treatment. This analysis period was determined in accordance with previously reported times of peak effect in rodents after i.p. administration (Barton et al., 2001; Klitgaard et al., 1998; Luszczki et al., 2003) and to exclude a potential anti-seizure effect of the solvent DMSO observed for 30 min after injection in experiments by our lab (Widmann et al., 2023). Post-treatment data were compared to a baseline period of 1 h before the treatment, excluding 5 min immediately before and after the injection to minimize the confounding influence of handling stress. Recordings with a generalized seizure during the post-treatment period were excluded from this analysis.

Using the fast Fourier transform (FFT) algorithm with a 10 s sliding Hanning window the ipsilateral hippocampal LFP signal power was calculated, and divided into the following frequency bands which are typical for preclinical rodent studies (Kadam et al., 2017): 1–4 Hz (delta), 4–8 Hz (theta), 8–13 Hz (alpha), 13–30 Hz (beta) and 30–80 Hz (gamma). In addition, the coastline, an aggregate of frequency and amplitude was computed (Lieb et al., 2018).

### 2.4 Nissl staining of frozen brain sections

After sacrificing mice by cervical dislocation, the brains were extracted and immediately snap-frozen in - 70 °C 2-methylbutane. 20 μm coronal sections were cut on a cryotome (Microm Cryo-Star HM 560, Thermo Fisher Scientific) and mounted onto poly-L-lysine coated glass slides (Menzel GmbH & Co KG). Nissl staining was performed based on the protocol of Paxinos et al. (Paxinos and Watson, 1986). After 10 min fixation in 4% PFA in 1× PBS the sections were dehydrated in a graded ethanol series (70%, 95%, 100%, 100%) and transferred to butyl acetate. Rehydration in a reverse ethanol series was followed by incubation in 0.5% cresyl violet solution for 20 min. Subsequently, the sections were again dehydrated in an increasing ethanol series and cleared in butyl acetate. Finally, the sections were embedded with Eukitt® mounting medium (ORSAtec GmbH) and glass coverslips. Images were taken with a Zeiss Axiophot microscope with a 2.5×/0.075 Plan-Neofluar objective connected to a AxioCam MRc5 camera (Carl Zeiss AG).

2 animals from the low-HPDs group (<50 s/h HPDs at 2 months), which were reinjected with KA after completion of the 2 months recordings of the development analysis, are neither included in any further LFP analysis nor in the morphological evaluation.

### 2.5 Data and statistical analysis

For statistical analysis GraphPad Prism version 9.5.1 for macOS was used. The development of epileptiform activity in female mice was analyzed with a 1-way mixed-effects model for repeated measures (recording time point as independent factor). The subgroup analysis was performed by fitting a 2-way mixed-effects model for repeated measures (high/low-HPDs group and recording time point as independent factors) and Tukey’s multiple comparisons test. For analyzing the effect of ASMs on epileptiform activity, a 2-way mixed-effects model for repeated measures (pre–/post-treatment period and ASMs as independent factors) followed by Šídák’s multiple comparisons test was applied. The occurrence of generalized seizures after ASMs treatments was analyzed with Chi-square test. Given the log-normal distribution of the power bands data, a log-transformation was performed prior to a 2-way repeated measures ANOVA (pre–/post-treatment period and ASMs as independent factors) followed by Šídàk’s multiple comparisons test. *P*-values smaller than 0.05 were considered statistically significant. Data are presented as mean ± standard deviation (SD), with data for individual animals also shown.

## 3 Results

### 3.1 Post-IHKA development of epileptiform activity in female mice

**Fig. 2:**
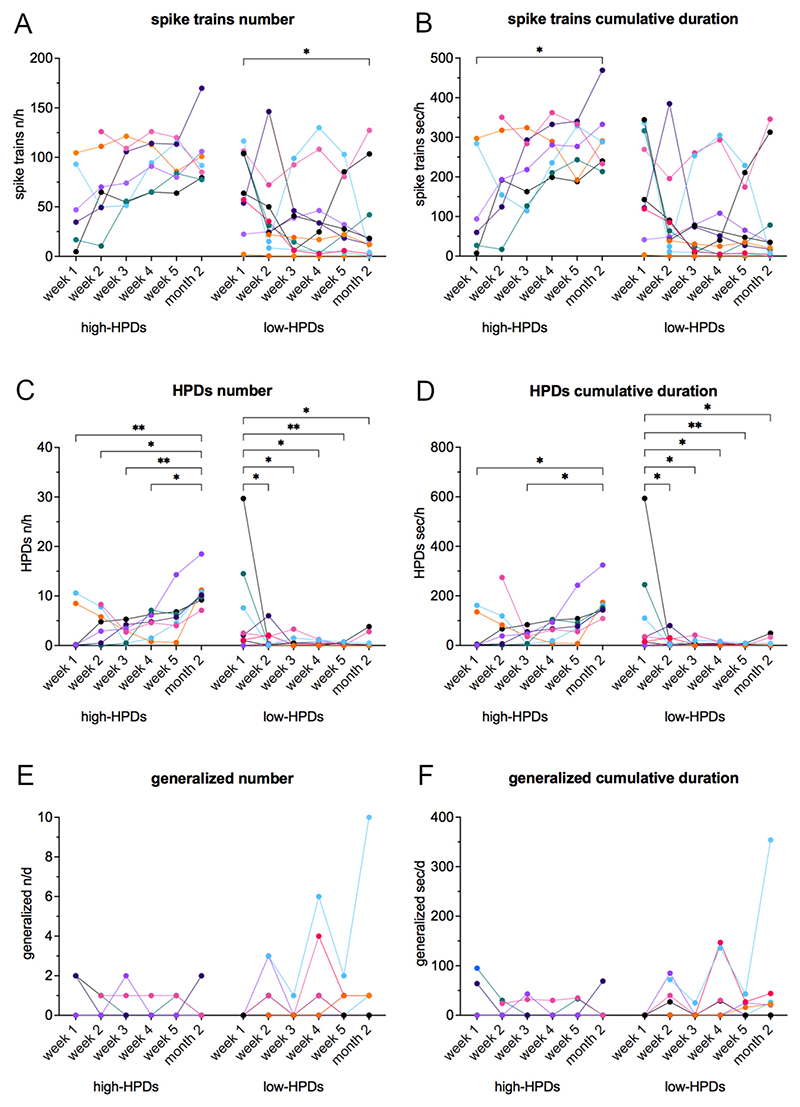
Development of epileptiform activity in the ipsilateral hippocampus in female mice after IHKA. (A-F) Separated into high-HPDs group (>50 s/h HPDs at 2 months, n = 7) and low-HPDs group (<50 s/h HPDs at 2 months, n = 11), (A) number [n/h] and (B) cumulative duration [s/h] of spike trains, (C) number [n/h] and (D) cumulative duration [s/h] of HPDs, (E) number [n/d] and (F) cumulative duration [s/d] of generalized seizures were assessed during weekly 24 h recordings for the first 5 weeks and at 2 months. Females of the high-HPDs group showed a significant increase of HPDs and a tendency to increase spike trains, while HPDs significantly decreased and spike trains tended to decrease in the low-HPDs group. Regarding generalized seizures, no significant differences were detected. Note one animal of the low-HPDs group with strikingly higher generalized seizure activity increasing up to 10 generalized seizures at the 2 months recording time point. Curves represent individual animals. Two-way mixed-effects model for repeated measures followed by Tukey’s multiple comparisons test (significance levels shown in graph). * p < 0.05; ** p < 0.01.

To evaluate the development of epileptiform activity, 18 female mice after IHKA injection were monitored with weekly 24 h recordings during the first 5 weeks and at the 2 months interval (Fig. 1 A). In general, female mice, with considerable inter-animal variability, presented the same type of epileptiform activity, namely spike trains, HPDs and generalized seizures (Fig. 1 C and D, Table 1), as previously described in male IHKA mice (Riban et al., 2002; Zangrandi et al., 2016). Statistical analysis of all recorded animals did not reveal any significant differences between the different recording time points. Based on the manifestation of ipsilateral HPDs at 2 months, 2 subgroups (>50 s/h HPDs termed high-HPDs group, <50 s/h HPDs termed low-HPDs group) were analyzed in detail (Fig. 2 A to F, Table 1). We consider mice with >50 s/h HPDs at 2 months as suitable to test potential antiepileptic interventions with a short half-life. These mice displayed stably established chronic focal epilepsy with sufficient HPDs to measure a potential reduction within a short period of time.

In females of the high-HPDs group (*n* = 7), spike trains in the ipsilateral hippocampus tended to increase (Fig. 2 A and B, Table 1), but statistical significance was reached only for the cumulative duration of spike trains (Fig. 2 B) at 2 months compared to week 1 (F(5, 74) = 4.74, 95% CI -304.5 to −15.62, *p* = 0.0212). Regarding ipsilateral HPDs, we observed an increase in the high-HPDs group during the post-surgery period: the number of HPDs (Fig. 2 C, Table 1) at 2 months was significantly increased compared to week 1 (2-way mixed-effects model for repeated measures (F(5, 90) = 5.53) followed by Tukey’s multiple comparisons test (95% CI -13.81 to −1.637, *p* = 0.0049)), week 2 (95% CI -12.52 to −0.8254, *p* = 0.0158), week 3 (95% CI -14.05 to −2.354, *p* = 0.0013) and week 4 (95% CI -12.36 to −0.6682, *p* = 0.0199); the cumulative duration of HPDs (Fig. 2 D, Table 1) was significantly higher at 2 months in comparison to week 1 (F(5, 90) = 4.21, 95% CI -239.1 to −4.243, *p* = 0.0378) and week 3 (95% CI -247.1 to −21.49, *p* = 0.0102).

Female mice of the low-HPDs group displaying <50 s/h HPDs at 2 months (*n* = 11), in contrast, showed a decrease of epileptiform activity in the ipsilateral hippocampus over time. Spike trains (Fig. 2 A and B, Table 1) displayed a tendency to decrease, but solely the number of spike trains (Fig. 2 A) at week 1 in comparison to 2 months (F(5, 74) = 4.29, 95% CI 1.260 to 79.58, *p* = 0.0391) reached statistical significance. HPDs showed a significant decrease in the low-HPDs group: the number of HPDs (Fig. 2 C, Table 1) was significantly higher at week 1 compared to week 2 (F(5, 90) = 5.53, 95% CI 0.6317 to 10.46, *p* = 0.0176), week 3 (95% CI 0.8859 to 10.94, *p* = 0.0116), week 4 (95% CI 0.9887 to 11.30, *p* = 0.0101), week 5 (95% CI 1.414 to 11.25, *p* = 0.0041) and 2 months (95% CI 0.9226 to 10.75, *p* = 0.0105); the cumulative duration of HPDs (Fig. 2 D, Table 1) was significantly increased at week 1 compared to week 2 (F(5, 90) = 4.21, 95% CI 7.969 to 197.7, *p* = 0.0256), week 3 (95% CI 11.69 to 205.6, *p* = 0.0189), week 4 (95% CI 11.70 to 210.7, *p* = 0.0193), week 5 (95% CI 19.14 to 208.9, *p* = 0.0092) as well as 2 months (95% CI 12.70 to 202.4, *p* = 0.0168).

Additional analysis of the contralateral hippocampal (Supplemental Table 2) and the cortical electrode (Supplemental Table 3) confirmed that HPDs remain mainly confined to the ipsilateral hippocampus as originally described in the paper of (Riban et al., 2002). At 2 months none of the animals showed >50 s/h HPDs in the contralateral hippocampus and only one animal of the high-HPDs group in the cortical electrode.

Regarding generalized seizures (Fig. 2 E and F), we did not detect any significant differences between the investigated time points. Mice of the high-HPDs group showed a maximum of 2 generalized seizures per day. 3 females of the high-HPDs group (43%) had exclusively focal epileptic activity without generalized seizures in our recordings, while 4 (57%) developed at least one generalized seizure during the whole observation period. In the low-HPDs group, 4 females (36%) did not show any generalized seizures, 7 (64%) had at least one generalized seizure throughout the recording period. Surprisingly, one female of the low-HPDs group showed strong generalized seizure activity, increasing up to 10 generalized seizures during 24 h at the 2 months recording time point.

### 3.2 Effect of ASMs in female IHKA mice

**Fig. 3:**
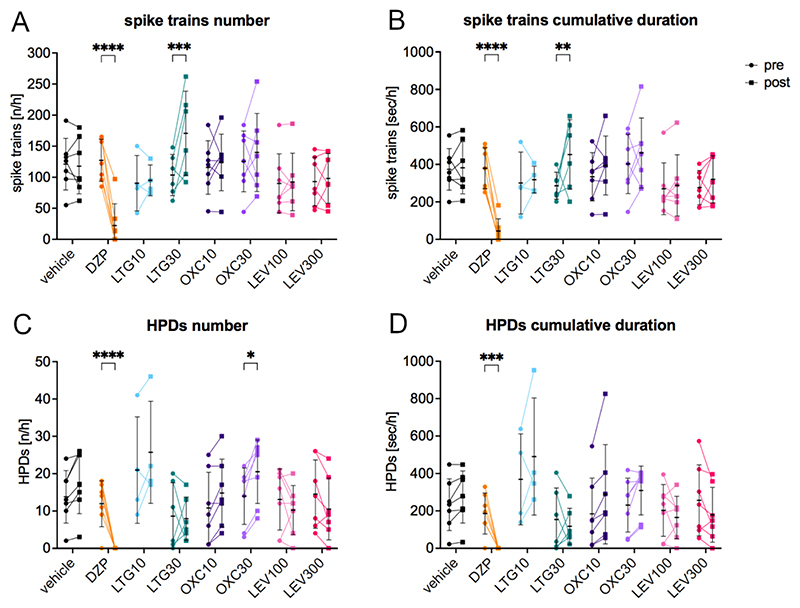
Effect of frequently prescribed new generation ASMs in female IHKA mice. (A) Number [n/h] and (B) cumulative duration [s/h] of spike trains and (C) number [n/h] and (D) cumulative duration [s/h] of HPDs 35–94 min after treatment compared to the 1 h pre-treatment period. The higher dose of LTG caused a significant increase in spike trains (number and cumulative duration) and the higher dose of OXC a significantly increased number of HPDs. The benzodiazepine DZP highly significantly reduced both spike trains and HPDs. Data (n = 7) were analyzed with a two-way linear mixed model for repeated measures followed by Šídák’s multiple comparisons test and are presented as individual values with mean ± SD also shown. DZP, diazepam; LTG, lamotrigine; OXC, oxcarbazepine; LEV, levetiracetam. * p < 0.05; ** p < 0.01; *** p < 0.001; **** p < 0.0001.

To evaluate the effect of frequently prescribed new generation ASMs, 7 female IHKA mice of the high-HPDs group were tested with LTG 10 and 30 mg/kg, OXC 10 and 30 mg/kg, LEV 100 and 300 mg/kg, DZP 2.5 mg/kg as positive control and vehicle (10% DMSO and 90% saline with 3% Tween 80). Number and cumulative duration of ipsilateral spike trains and HPDs between 35 and 94 min after the treatment were compared to a baseline period corresponding to the last hour before the treatment (Fig. 3). Animals with generalized seizures during the post-treatment period were not included in the analysis of spike trains and HPDs due to the post-ictal depression of LFP activity, resulting in an uneven number of analyzed animals.

None of the tested ASMs showed a decreasing effect on epileptiform activity. On the contrary, the higher dose of LTG (30 mg/kg) caused a statistically significant increase in both number (Fig. 3 A; 170.7 ± 67.7, two-way linear mixed model for repeated measures (F(7, 34) = 15.66) followed by Šídák’s multiple comparisons test (95% CI -112.40 to −27.07, *p* = 0.0002)) and cumulative duration of spike trains (Fig. 3 B; 452.0 ± 185.8, F(7, 34) = 12.75, 95% CI –-318.40 to −30.16, *p* = 0.0098) and the higher dose of OXC (30 mg/kg) resulted in an increased number of HPDs (Fig. 3 C; 20.6 ± 8.5, F(7, 34) = 8.83, 95% CI -12.62 to −0.52, *p* = 0.0261) compared to the pre-treatment period (LTG30 number of spike trains 103.5 ± 33.1, LTG30 cumulative duration of spike trains 285.3 ± 72.3, OXC30 number of HPDs 14.0 ± 7.6). Intriguingly, we also observed a significantly increased occurrence of generalized seizures within 94 min after LTG (3 out of 7 animals after 10 mg/kg and 1 out of 7 after 30 mg/kg; Chi-square *p* = 0.0160). On the other hand, none of the animals developed generalized seizures following the other treatments. As expected, DZP significantly reduced both the number (Fig. 3 A; 22.4 ± 34.9, F(7, 34) = 15.66, 95% CI 64.80 to 144.90, *p* ≤0.0001) and the cumulative duration of spike trains (Fig. 3 B; 43.7 ± 65.6, F(7, 34) = 12.75, 95% CI 199.80 to 469.90, *p* ≤0.0001) in comparison to the pre-treatment period (number of spike trains 127.3 ± 33.8; cumulative duration of spike trains 378.5 ± 109.4). HPDs (Fig. 3 C and D; number 0.0 ± 0.0, F(7, 34) = 8.83, 95% CI 5.95 to 18.05, *p* ≤0.0001, cumulative duration 0.0 ± 0.0, F(7, 34) = 6.02, 95% CI 66.48 to 306.10, *p* = 0.0005) were completely eliminated after DZP in comparison to pre-treatment (number of HPDs 12.0 ± 6.2, cumulative duration of HPDs 186.3 ± 109.5).

**Fig. 4:**
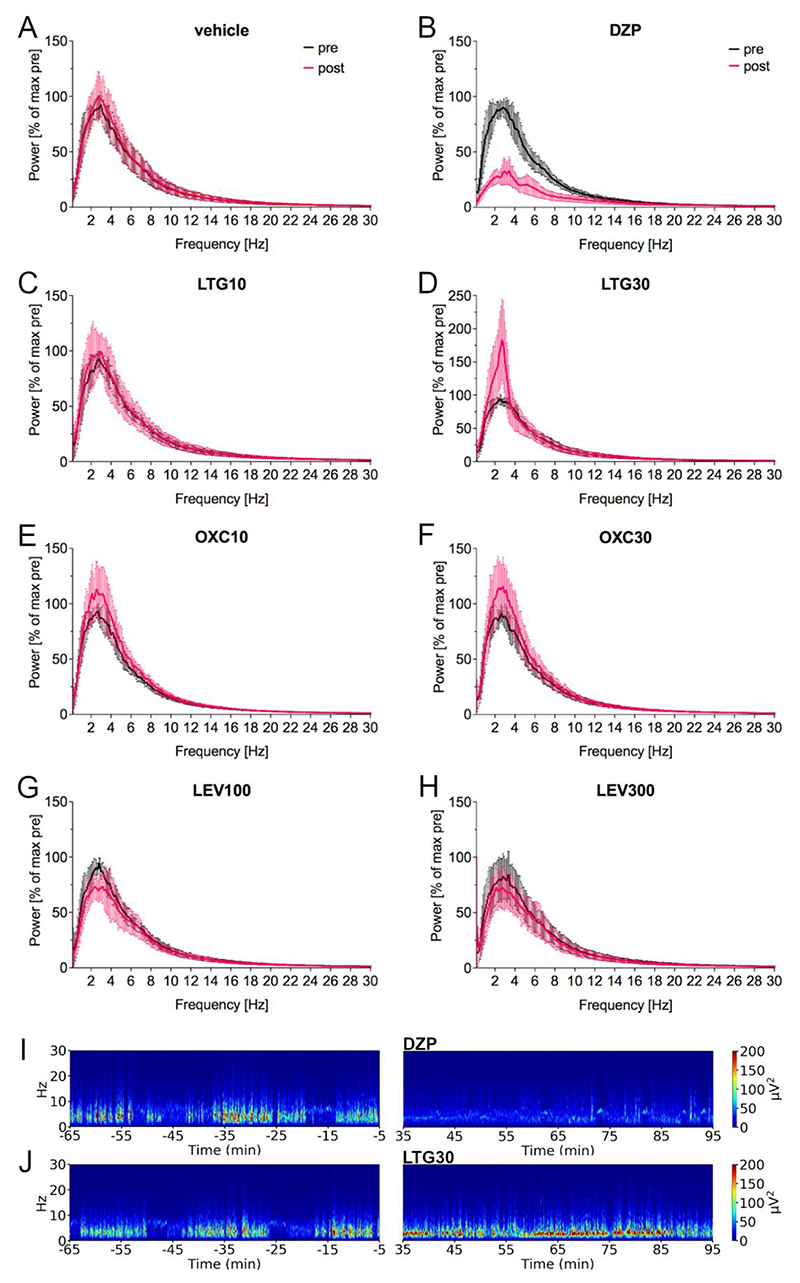
Power spectral analysis of the effect of frequently prescribed new generation ASMs in female IHKA mice. (A-H) Power spectral analysis showed (B) after DZP a marked decrease over a wide frequency range (delta, theta and alpha) and (D) after LTG30 a pronounced narrow increase in the delta frequency range (1–4 Hz). Note the different y-axis range for LTG30. Power in μV^2^/Hz in 5 s was calculated for the 64–5 min before (black) and 35–94 min after the treatment (magenta), normalized to the maximum of the pretreatment period [% of pretreatment maximum] and plotted as mean (line) ± SD (shaded area). (I-J) Representative spectrograms before and after (I) DZP showing a reduction and after (J) LTG30 with a narrow increase in the 1–4 Hz frequency range. DZP, diazepam; LTG, lamotrigine; OXC, oxcarbazepine; LEV, levetiracetam.

**Fig. 5:**
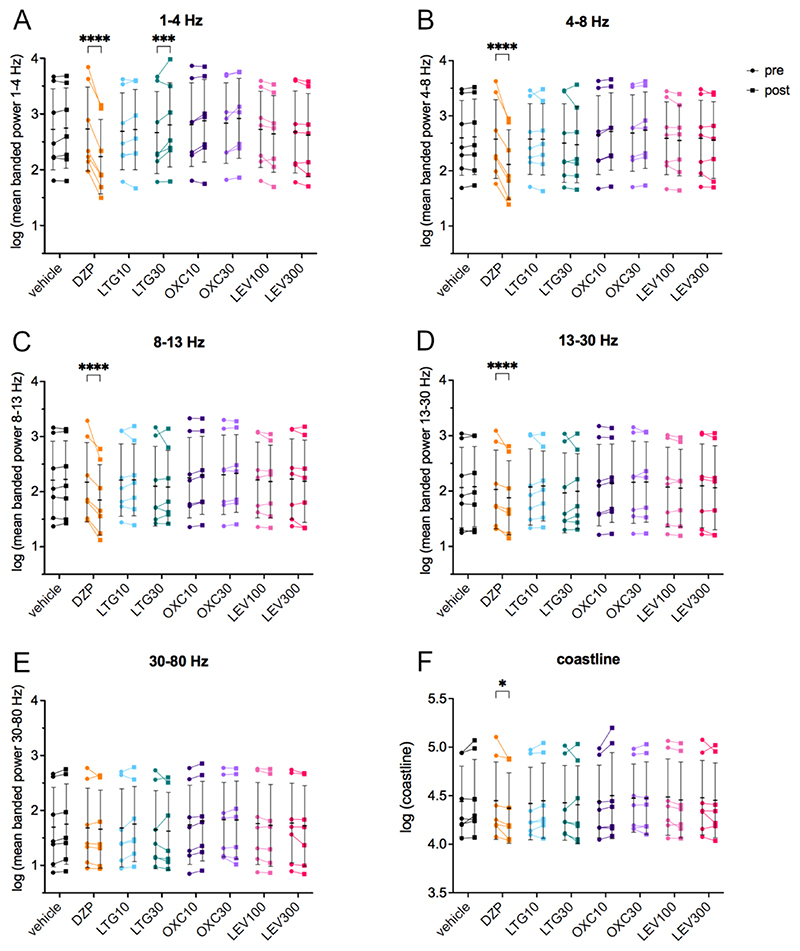
LFP signal power in different frequency bands after widely prescribed new generation ASMs in the female IHKA mouse model of TLE. (A) Mean power [μV2/Hz in 5 s] in the 1–4 Hz, (B) 4–8 Hz, (C) 8–13 Hz, (D) 13–30 Hz and (E) 30–80 Hz frequency bands as well as (F) the coastline in the 35–94 min after treatment were compared to the 1 h pre-treatment period, after performing a log-transformation. LTG30 caused a significant increase in the 1–4 Hz band, DZP significantly decreased the 1–4 Hz, 4–8 Hz, 8–13 Hz and 13–30 Hz bands as well as the coastline. Log-transformed data (n = 7) were analyzed with a two-way repeated measures ANOVA followed by Šídák’s multiple comparisons test and are presented as individual values with mean ± SD also shown. Note the different y-axis range for the coastline. DZP, diazepam; LTG, lamotrigine; OXC, oxcarbazepine; LEV, levetiracetam. * p < 0.05; *** p < 0.001; **** p < 0.0001.

Power spectral analysis (Fig. 4) indicated that LTG 30 mg/kg resulted in a pronounced narrow increase in the delta frequency range in the analyzed period of 35–94 min post-injection compared to the pre-treatment period (Fig. 4 D; representative spectrogram Fig. 4 J). In accordance, power in the 1–4 Hz frequency band (35–94 min after treatment compared to the pre-treatment period after log-transformation; Fig. 5 A, Table 2) increased statistically significantly following LTG 30 mg/kg (two-way repeated measures ANOVA (F (7, 42) = 44.00) followed by Šídák’s multiple comparisons test (95% CI -0.22 to −0.05, *p* = 0.0003)) in comparison to the pre-treatment period. DZP (Fig. 4 B, representative spectrogram Fig. 4 I) caused a marked reduction over a wide frequency range (delta, theta and alpha) of the power spectrum. Accordingly, the 1–4 Hz (F(7, 42) = 44.00, 95% CI 0.41 to 0.58, p ≤0.0001; Fig. 5 A, Table 2), 4–8 Hz (F(7, 42) = 31.38, 95% CI 0.37 to 0.55, p ≤0.0001; Fig. 5 B), 8–13 Hz (F(7, 42) = 11.20, 95% CI 0.22 to 0.42, p ≤0.0001; Fig. 5 C) and the 13–30 Hz (F(7, 42) = 4.27, 95% CI 0.07 to 0.23, p ≤0.0001; Fig. 5 D) frequency band revealed a statistically significant reduction in comparison to the pre-treatment baseline. Furthermore, the coastline (Fig. 5 F, Table 2), an aggregate of amplitude and frequency, was significantly reduced after DZP (F(7, 42) = 3.22, 95% CI 0.01 to 0.15, *p* = 0.0276) compared to pre-treatment.

### 3.3 Morphological evaluation of female IHKA mice

**Fig. 6:**
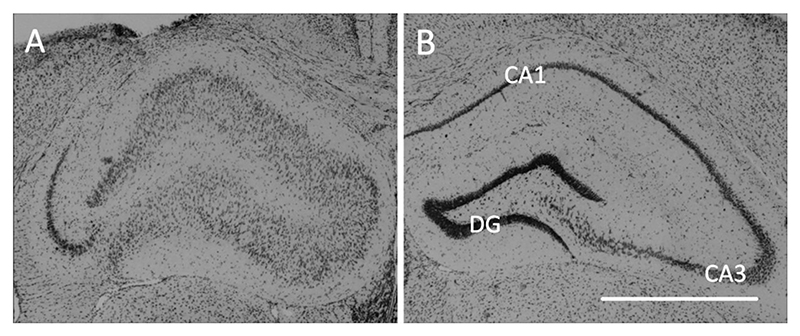
Ipsilateral neuropathological changes after IHKA in female mice. (A) Representative microscope image showing the characteristic pattern of ipsilateral morphological alterations with disintegration of the normal hippocampal layer structure, characterized by extensive neuronal loss in CA1 and less pronounced in CA3 as well as dispersion of granule cells, in a 20 μm Nissl-stained section of the dorsal hippocampus near the injection site (RC -1.8 mm). The tissue damage above the hippocampus is caused by removal of the depth electrode. (B) In the contralateral non-injected hippocampus layer integrity is mostly preserved. CA, cornu ammonis; DG, dentate gyrus; Scale bar = 1 mm.

Nissl-stained brain sections (*n* = 18) were analyzed after the experimental series with ASMs to assess morphological alterations related to hippocampal sclerosis (Fig. 6). All mice of the high-HPDs group (n = 7) displayed typical neuropathological changes of the KA-injected hippocampus (Supplemental Fig. 1) with massive dispersion of the granule cells and marked loss of Nissl-stained cells mainly in CA1 and partially in CA3. In addition, the same morphological changes were present in mice from the low-HPDs group which developed generalized seizures (n = 7). Of the remaining animals from the low-HPDs group showing neither sufficient HPDs nor generalized seizures (*n* = 4), one did not develop any of the typical morphological alterations, one did show CA1 and CA3 cell loss as well as granule cell dispersion despite the absence of epileptiform LFP activity and two were excluded because they had been reinjected with KA.

## 4 Discussion

Women with epilepsy face special challenges (for review see (Harden and Pennell, 2013; Stephen et al., 2019)) and therefore a well-characterized female epilepsy model is crucial for the development of novel therapeutic strategies tailored to gender-specific needs. However, after a decades-long focus on males in preclinical animal studies, females have only recently moved into the center of scientific interest. In the IHKA mouse model of TLE, one of the most extensively studied models in males (Bouilleret et al., 2000; Riban et al., 2002; Suzuki et al., 1995), recent studies suggest sex-specific differences regarding development and manifestation of seizures (Cutia et al., 2023a; Li et al., 2020; Twele et al., 2016). The latest lines of research mainly focused on investigating mechanisms underlying reproductive endocrine comorbidities associated with TLE (Cutia et al., 2022, 2023b; Ingram et al., 2022; Li and Christian-Hinman, 2022). Importantly, however, drug-responsiveness of HPDs (in male mice a well-accepted model of drug-resistant seizures for screening of novel drugs (Kehne et al., 2017)) has not yet been investigated in females.

In this study, we characterized the IHKA model in female C57BL/6 N mice regarding the development of seizures, hippocampal neuropathological alterations and in particular the effect of ASMs on HPDs and on the power spectrum.

Within 2 months after IHKA, female mice developed epileptiform activity (spike trains, HPDs and generalized seizures) as we previously observed in male IHKA mice (Zangrandi et al., 2016). It is particularly noteworthy that female IHKA mice in our study established HPDs, which had been validated as a model of drug-resistant focal seizures in male mice (Duveau et al., 2016; Riban et al., 2002; Zangrandi et al., 2016). This contrasts with the findings of Twele et al. describing HPDs frequently in males, but rarely in females of various mouse strains, including C57BL/6 mice (Twele et al., 2016). These discrepant findings could be attributed to substrain differences, since a strikingly different sensitivity to the chemoconvulsant pilocarpine has been reported (Bankstahl et al., 2012; Müller et al., 2009). We used C57BL/6 N mice as in our previous studies with males, while Twele et al. did not specify the substrain. Differing definitions for HPDs also contribute to the observed discrepancy. In our study, HPDs were defined as series of spikes lasting ≥10 s with a minimum of 1.33 Hz, in accordance to our previous studies in males. Twele et al. applied a more stringent definition of HPDs requiring a longer duration of ≥20 s and a higher frequency of 10–20 Hz. Several recent studies define HPDs with a minimum duration of 5 s and a minimum frequency of 5 Hz (Cutia et al., 2023a; Zeidler et al., 2018). Analyzing our dataset according to these definitions of HPDs, we did not detect HPDs in female IHKA mice. This is due to a peak of spike frequency between 3 and 4 Hz (see also Fig. 4 for the power spectra) in our experiments. This might reflect a strain difference between C57BL/6 N (used in this study and C57BL/6 J used in other studies (Cutia et al., 2023a; Duveau et al., 2016; Zeidler et al., 2018). Therefore, considerations on drug resistance need to consider potential strain influences on the model. Overall, a standardization of the definitions would be needed to facilitate the comparability of different studies. Regarding generalized seizures, our observations of an average number of <1 per day are in line with the data of Twele et al. Furthermore, these authors described a faster onset of seizures without a clear seizure-free latent period in the females in contrast to the males showing a latent period of about 2 weeks. In line with this, a majority of mice in our study presented HPDs already 1 week after IHKA. Since we consider mice with >50 s/h HPDs at 2 months as having stably established chronic focal epilepsy and suitable to test potential therapeutic interventions, we divided our mice into two subgroups. Females with sufficient HPDs (>50 s/h at 2 months) showed increasing epileptiform activity during the post-IHKA observation period. In contrast, animals that did not develop enough HPDs (<50 s/h at 2 months) displayed a more variable development with a decrease in HPDs and spike trains and a predominance of generalized seizures in one individual.

The IHKA mouse model has been integrated into the current epilepsy therapy screening program of the NIH, given the high predictive value of HPDs in screening novel drugs (Kehne et al., 2017). In previous studies of our lab, a HPD cutoff of >50 s/h has proven effective for identifying animals suitable for testing drugs with comparably short half-lives. Excluding mice with <50 s/h spent in HPDs narrows the focus of the study, but animals displaying too little seizure-like activity result in a high variability in short-term experiments due to the short half-life of the drugs. Including animals with less HPDs would require prolonged treatment either through multiple injections or osmotic pump implantation. Assessing the effect of drugs on infrequent generalized seizures, such as in 7 of 11 animals in the low-HPDs group in the present study, would undoubtedly be of interest regarding clinical translatability. However, since this would necessitate an elaborate experimental design with repeated drug administrations over extended periods and long-term recordings, it remains to be investigated in future studies. However, for evaluating treatments with long-term effects, such as gene therapy, also animals exclusively experiencing generalized seizures have been included in prior studies (Agostinho et al., 2019).

We acknowledge that the use of a discontinuous recording system constitutes a significant limitation of the present study when analyzing the development of epileptiform activity in particular regarding infrequently occurring generalized seizures. However, our system enables 4 channel recordings extending over several months by using detachable Neurologgers that can be equipped with fresh batteries for each recording cycle. This extended 4 channel recording capability is paramount for our focus on analyzing treatments after the animals have developed a substantial number of HPDs, which typically occurs after approximately 2 months. Nevertheless, future research could benefit from employing continuous recording systems to explore circadian or other rhythmic patterns in seizure distribution.

Moreover, we showed that female IHKA mice replicate neuropathological alterations as described in human TLE, consistent with previous studies in males (Bouilleret et al., 1999; Riban et al., 2002) and the findings of Cutia et al. in female C57BL/6 J mice (Cutia et al., 2023a). Specifically, we observed ipsilateral hippocampal neurodegeneration, mainly in CA1 and less frequently in CA3, accompanied by extensive granule cell dispersion in all animals with HPDs and/or generalized seizures.

While in male mice HPDs after IHKA were described as a model of drug-resistant seizures (Duveau et al., 2016; Riban et al., 2002; Zangrandi et al., 2016), the only previous study testing ASMs in female IHKA mice did not quantify HPDs, but instead so-called seizure-like events (SLEs) with a duration ≥3 s (Klein et al., 2015). Since drug-responsiveness of HPDs in female IHKA mice has not been addressed yet, we sought to test LTG, OXC and LEV, which are listed among the most frequently prescribed ASMs over the last decade (Hochbaum et al., 2022). Of each of these ASMs we tested a dose effective in rodent seizure models (Barton et al., 2001; Klitgaard et al., 1998; Luszczki et al., 2003) and comparable to the therapeutic range for epilepsy patients, as well as a threefold higher dose, consistent with previous dosages used to evaluate drug-resistance of HPDs in male IHKA mice (Zangrandi et al., 2016). As a positive control we applied the benzodiazepine DZP, which had been shown to effectively suppress both drug-resistant HPDs in male (Duveau et al., 2016; Riban et al., 2002; Zangrandi et al., 2016) and seizure-like events in female IHKA mice (Klein et al., 2015). HPDs in the female IHKA mice did not respond to the tested ASMs, which is in line with prior studies in males (Duveau et al., 2016; Zangrandi et al., 2016). On the contrary, the higher dose of OXC even resulted in an increase in the number of HPDs. After the higher dose of 30 mg/kg LTG, we observed a significant increase in spike trains. Interestingly, Duveau et al. described in male IHKA mice a biphasic effect of LTG with an increase in HPDs after the same dose of 30 mg/kg, while even higher doses caused a decrease (Duveau et al., 2016). Since the broader definition of HPDs (≥5 s) in their study incorporates parts of the events that we count as spike trains, our findings of increased spike trains after 30 mg/kg LTG can be considered in line with their results. Intriguingly, we observed a significantly increased occurrence of generalized seizures after LTG, whereas no generalized seizures occurred in the other treatment groups. Seizure aggravation after LTG is known from patients with juvenile myoclonic epilepsy (Gesche et al., 2021), but to our knowledge has not been described in TLE patients. We additionally analyzed SLEs according to the criteria of Klein et al. (Klein et al., 2015) and found that in line with their results solely DZP significantly reduced both number and duration of SLEs (Supplemental Fig. 2).

To evaluate the effect of ASMs on the power of different frequency bands we performed power spectral analysis using FFT, which enabled analyzing the whole data set of 7 animals irrespective of generalized seizures. Since animals with generalized seizures are excluded from the analysis of spike trains and HPDs due to post-ictal depression of LFP activity, focusing on power spectral analysis could contribute to reducing the numbers of animals needed in accordance with the 3R concept, when evaluating drugs potentially facilitating generalized seizures causing higher drop-out rates. Surprisingly, the higher dose of LTG resulted in a pronounced narrow increase in the power in the low frequency range (1–4 Hz corresponding to delta). However, the higher frequency bands of the beta and gamma range associated with generalized seizures (Puttachary et al., 2015; Tse et al., 2014) appeared unaffected after LTG, most likely because generalized seizures are, despite the observed increased occurrence, still too rare to influence the power spectrum of the whole analysis period.

DZP, as expected, significantly reduced spike trains and completely abolished HPDs. This was further corroborated by a decrease over a wide range of the power spectrum, with a significant reduction of the 1–4 Hz, 4–8 Hz, 8–13 Hz and 13–30 Hz bands as well as the coastline. Benzodiazepines, in general, have shown broad-spectrum antiseizure efficacy in various animal models as well as epilepsy patients upon acute administration but lose their effect when given chronically, which precludes their application for long-term epilepsy treatment (Riss et al., 2008) but renders them a suitable positive control to confirm that epileptiform activity can be attenuated.

A potential confounding influence of estrous cycle stages on baseline seizure activity in the ASMs experiment was compensated by comparing post-treatment data to the respective pre-treatment baseline data of the same day. Regarding the effect of estrous cycle stage during kainic acid injection on seizure susceptibility, a previous study showed higher susceptibility to acute seizures induced by i.p. kainic acid during estrus compared to diestrus, characterized by a shorter latency to onset and a longer duration of seizures (Maguire et al., 2005). However, a long-term effect on epileptogenesis and manifestation of epilepsy in the IHKA model has not been investigated yet. Either estrous cycle stages were not analyzed (Twele et al., 2016) or IHKA injections were conducted on diestrus (Cutia et al., 2023a; Li et al., 2020). We performed a basic analysis of the estrous cycle stage by vaginal lavage on the day of IHKA (Supplemental Table 1). However, since our main focus of interest was on analyzing the influence of ASMs on HPDs and the power spectrum, we refrained from a detailed estrous cycle characterization, which would require invasive procedures such as measurements of hormone levels and higher numbers of animals due to the short duration of certain estrous cycle stages.

Overall, HPDs in female IHKA mice were refractory to established doses of frequently used current ASMs but suppressed by the benzodiazepine DZP, which is in line with the previously reported drug-refractoriness of HPDs in male IHKA mice (Duveau et al., 2016; Riban et al., 2002; Zangrandi et al., 2016). In this regard, HPDs in female IHKA mice fulfill the requirements for a model of pharmaco-resistant seizures poorly responding to at least two current ASMs (Stables et al., 2003) and are suitable for the screening of novel ASM candidates.

## 5 Conclusions

In conclusion, we confirmed that female mice after IHKA reproduce essential LFP and morphological features of human TLE patients that were previously modeled in male IHKA mice, in particular drug-resistance of HPDs. Importantly, this is the first study in female IHKA mice evaluating the influence of widely prescribed ASMs as well as DZP on HPDs and different frequency bands of the power spectrum. Considering the special issues complicating the therapeutic management of epilepsy in women, inclusion of females in the quest for novel treatment strategies is imperative and has in recent years been demanded by funding agencies such as the NIH (Clayton and Collins, 2014). A well-characterized female model is a prerequisite for the development of novel therapeutic strategies tailored to sex-specific needs and for studies on the effect of epilepsy and ASMs during pregnancy.

## Supplementary Material

Supplementary data

## Figures and Tables

**Table 1:** Development of epileptiform activity in the ipsilateral hippocampus in female mice after IHKA presented as mean ± standard deviation.

All animals	week 1	week 2	week 3	week 4	week 5	2 months
Spike trains number [n]	62.0 ± 40.3	50.6 ± 41.7	55.0 ± 39.4	64.6 ± 47.1	59.1 ± 42.1	58.4 ± 52.3
Spike trains duration [s]	164.4 ± 129.0	129.7 ± 120.6	137.9 ± 112.7	171.4 ± 134.1	152.2 ± 124.4	162.6 ± 154.8
HPDs number [n]	5.2 ± 8.2	2.3 ± 2.9	1.5 ± 1.8	2.2 ± 2.6	2.5 ± 3.9	4.7 ± 5.7
HPDs duration [s]	90.3 ± 158.1	41.0 ± 68.5	20.4 ± 24.9	31.6 ± 39.6	37.7 ± 63.0	72.6 ± 93.0
High-HPDs	**week 1**	**week 2**	**week 3**	**week 4**	**week 5**	**2** **months**
Spike trains number [n]	50.1 ± 40.6	68.8 ± 39.2	81.7 ± 29.7	95.5 ± 24.0	94.5 ± 21.5	101.5 ± 31.9
Spike trains duration [s]	128.4 ± 129.3	192.9 ± 113.6	217.7 ± 85.0	272.9 ± 61.4	271.9 ± 65.6	295.4 ± 86.9
HPDs number [n]	3.3 ± 4.9	4.3 ± 3.3	2.8 ± 1.8	4.5 ± 2.4	6.0 ± 4.2	11.0 ± 3.6
HPDs duration [s]	51.1 ± 76.1	84.0 ± 93.6	38.5 ± 27.3	65.4 ± 38.6	93.1 ± 73.2	172.8 ± 69.9
Low-HPDs	**week 1**	**week 2**	**week 3**	**week 4**	**week 5**	**2** **months**
Spike trains number [n]	70.0 ± 40.5	39.1 ± 40.7	36.3 ± 35.1	40.6 ± 47.4	36.5 ± 35.9	30.9 ± 43.7
Spike trains duration [s]	188.4 ± 130.6	89.4 ± 111.4	82.1 ± 96.7	92.4 ± 121.9	76.0 ± 85.6	78.0 ± 126.5
HPDs number [n]	6.5 ± 9.9	1.0 ± 1.8	0.6 ± 1.1	0.4 ± 0.5	0.2 ± 0.3	0.7 ± 1.3
HPDs duration [s]	116.4 ± 195.4	13.6 ± 24.4	7.8 ± 13.3	5.2 ± 6.9	2.4 ± 3.3	8.9 ± 16.7

**Table 2:** LFP signal power in different frequency bands after ASMs in female IHKA mice presented as mean ± standard deviation.

			1–4 Hz			4–8 Hz			8–13 Hz			13–30 Hz			coastline		
			pre	post		pre	post		pre	post		pre	post		pre	post	
	vehicle		2.72 ± 0.73	2.75 ± 0.72		2.60 ± 0.68	2.62 ± 0.68		2.21 ± 0.71	2.23 ± 0.70		2.07 ± 0.73	2.08 ± 0.72		4.44 ± 0.36	4.48 ± 0.39	
	DZP		2.73 ± 0.75	2.23 ± 0.67		2.58 ± 0.72	2.12 ± 0.63		2.18 ± 0.72	1.85 ± 0.64		2.03 ± 0.71	1.88 ± 0.67		4.45 ± 0.40	4.37 ± 0.36	
	LTG10		2.69 ± 0.69	2.72 ± 0.72		2.58 ± 0.64	2.57 ± 0.65		2.21 ± 0.66	2.22 ± 0.65		2.08 ± 0.69	2.10 ± 0.63		4.42 ± 0.37	4.45 ± 0.39	
	LTG30		2.66 ± 0.74	2.80 ± 0.75		2.51 ± 0.71	2.48 ± 0.69		2.10 ± 0.72	2.09 ± 0.66		1.97 ± 0.72	2.00 ± 0.68		4.43 ± 0.39	4.41 ± 0.40	
	OXC10		2.81 ± 0.75	2.88 ± 0.74		2.65 ± 0.72	2.72 ± 0.70		2.26 ± 0.73	2.30 ± 0.71		2.11 ± 0.74	2.15 ± 0.71		4.44 ± 0.37	4.50 ± 0.44	
	OXC30		2.83 ± 0.72	2.92 ± 0.72		2.69 ± 0.70	2.74 ± 0.70		2.31 ± 0.72	2.33 ± 0.71		2.16 ± 0.74	2.17 ± 0.73		4.48 ± 0.35	4.48 ± 0.37	
	LEV100		2.72 ± 0.68	2.64 ± 0.69		2.59 ± 0.66	2.55 ± 0.64		2.22 ± 0.69	2.19 ± 0.66		2.08 ± 0.72	2.06 ± 0.70		4.49 ± 0.39	4.46 ± 0.39	
	LEV300		2.68 ± 0.74	2.62 ± 0.74		2.59 ± 0.69	2.56 ± 0.70		2.23 ± 0.73	2.19 ± 0.75		2.10 ± 0.76	2.06 ± 0.76		4.48 ± 0.38	4.45 ± 0.39	

## Data Availability

The data that support the findings of this study are available from the corresponding author upon reasonable request.
