# Supplementary material for: Characterization of the intrahippocampal kainic acid model in female mice with a special focus on seizure suppression by antiseizure medications
Source: Exp Neurol. Author manuscript; Available in PMC 2024 Jun 1. (PMC7615823; doi:10.1016/j.expneurol.2024.114749)
Supplement: Supplementary data [file EMS194709-supplement-Supplementary_data.docx]

APPENDIX

***
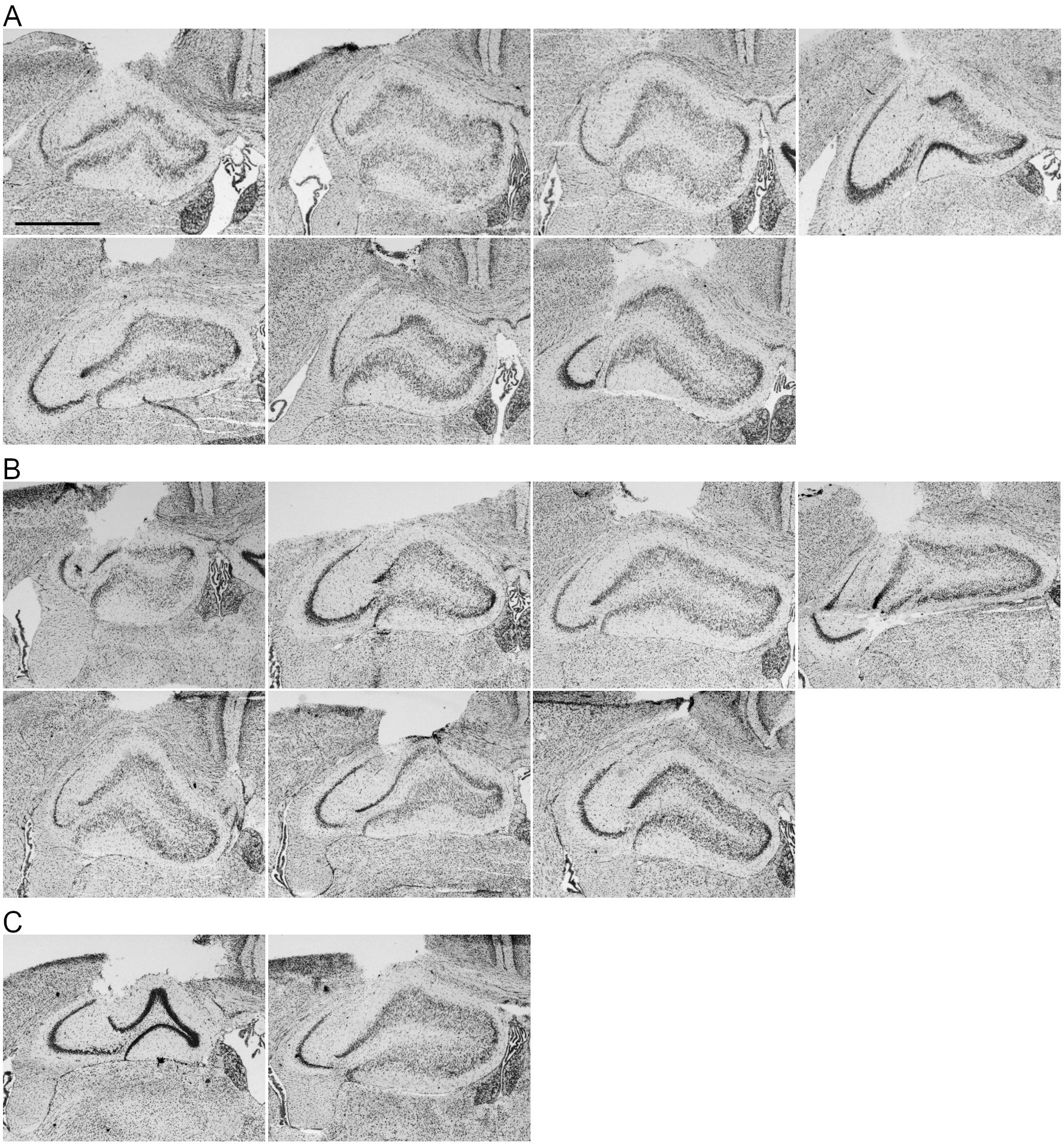
***

***Supplemental Figure 1. Overview of Nissl-stained sections for analysis of ipsilateral neuropathological changes after IHKA in female mice.***

*(A) Microscope images of mice from the high-HPDs group (n = 7) and (B) from the low-HPDs group with generalized seizures (n = 7) showing dispersion of granule cells and loss of Nissl-stained cells mainly in CA1.*

*(C) Microscope images of mice with <50 s/h HPDs and no generalized seizures (n = 2), the left one without typical morphological alterations and the right one displaying granule cell dispersion and neuronal loss in CA1.*

*20 µm Nissl-stained sections of the dorsal hippocampus near the KA injection site. Images were taken with a 2.5x/0.075 objective. Scale bar = 1mm*


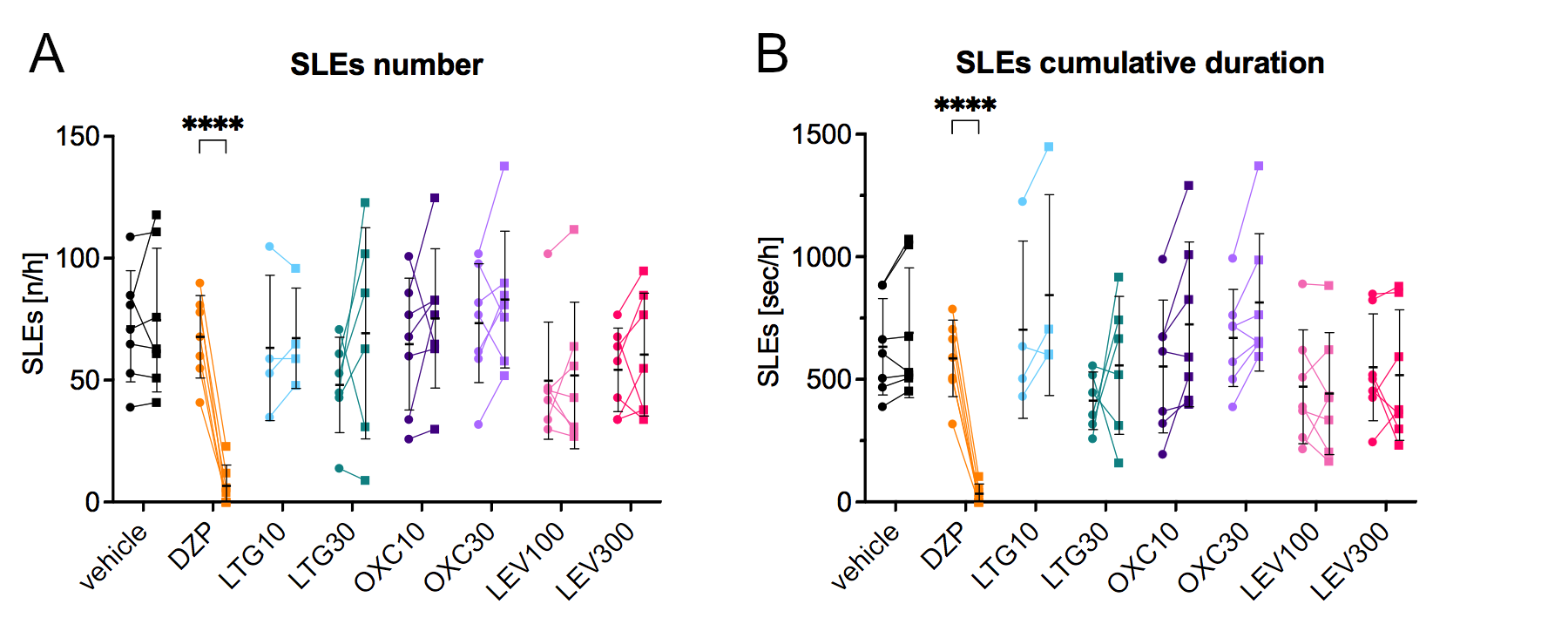


**Supplemental Figure 2. Effect of ASMs on seizure-like events (SLEs) 30-90 min after treatment.** SLEs were defined according to the criteria of (Klein et al. 2015) as events with a duration >3 s and a frequency of >1 Hz. DZP caused a significant reduction of number (6.4 ± 8.5, F (7, 34) =12.24, 95 % CI 36.63 to 85.65, p < 0.0001) and duration of SLEs (30.9 ± 39.9, F (7, 34) = 13.73, 95 % CI 352.7 to 751.6, p < 0.0001) compared to pre-treatment (number of SLEs 67.6 ± 16.9, duration of SLEs 583.0 ± 156.2). Data (n = 7) were analyzed with a two-way linear mixed model for repeated measures followed by Šídák’s multiple comparisons test and are presented as individual values with mean ± SD also shown. DZP, diazepam; LTG, lamotrigine; OXC, oxcarbazepine; LEV, levetiracetam. **** p < 0.0001

|  | diestrus | non-diestrus |
| --- | --- | --- |
| high-HPDs | 6 | 1 |
| low-HPDs generalized | 3 | 4 |
| low-HPDs no generalized | 3 | 1 |

***Supplemental Table 1. Estrous cycle stage at time of IHKA.***

*Estrous cycle stage was determined based on vaginal cytology obtained by vaginal lavage (Gonzalez, 2016) with 0.9 % saline on the day of IHKA injection followed by Giemsa staining.*

| high-HPDs | week 1 | week 2 | week 3 | week 4 | week 5 | 2 months |
| --- | --- | --- | --- | --- | --- | --- |
| Spike trains number [n] | 14.4 ± 23.1 | 22.2 ± 17.5 | 28.6 ± 28.2 | 26.0 ± 29.8 | 21.8 ± 24.7 | 16.6 ± 20.9 |
| Spike trains duration [s] | 29.9 ± 50.9 | 54.0 ± 61.5 | 72.6 ± 78.7 | 66.8 ± 83.8 | 57.2 ± 70.4 | 42.8 ± 55.1 |
| HPDs number [n] | 0.5 ± 1.0 | 2.8 ± 6.8 | 2.2 ± 4.9 | 1.1 ± 2.2 | 1.0 ± 1.7 | 0.9 ± 1.3 |
| HPDs duration [s] | 7.9 ± 16.4 | 47.9 ± 120.1 | 33.1 ± 74.9 | 17.6 ± 38.3 | 15.7 ± 28.5 | 13.7 ± 19.1 |
| low-HPDs | **week 1** | **week 2** | **week 3** | **week 4** | **week 5** | **2 months** |
| Spike trains number [n] | 6.9 ± 13.1 | 13.5 ± 20.1 | 12.2 ± 16.6 | 3.3 ± 4.2 | 7.7 ± 13.3 | 7.8 ± 10.6 |
| Spike trains duration [s] | 14.3 ± 33.0 | 34.2 ± 55.9 | 27.9 ± 41.3 | 5.0 ± 6.6 | 13.5 ± 23.0 | 15.5 ± 22.2 |
| HPDs number [n] | 0.2 ± 0.6 | 0.4 ± 0.6 | 0.5 ± 1.1 | 0.1 ± 0.1 | 0.0 ± 0.1 | 0.1 ± 0.2 |
| HPDs duration [s] | 2.8 ± 8.3 | 5.1 ± 7.6 | 9.6 ± 21.1 | 1.1 ± 1.9 | 0.6 ± 1.1 | 1.0 ± 2.6 |

***Supplemental Table 2. Analysis of the contralateral electrode in female mice after IHKA presented as mean ± standard deviation.***

| high-HPDs | week 1 | week 2 | week 3 | week 4 | week 5 | 2 months |
| --- | --- | --- | --- | --- | --- | --- |
| Spike trains number [n] | 8.0 ± 5.1 | 8.3 ± 4.7 | 5.5 ± 3.8 | 3.9 ± 3.2 | 6.1 ± 4.8 | 7.2 ± 9.0 |
| Spike trains duration [s] | 18.8 ± 14.3 | 16.9 ± 14.8 | 8.2 ± 6.5 | 6.4 ± 5.9 | 8.6 ± 7.2 | 13.9 ± 22.7 |
| HPDs number [n] | 0.3 ± 0.4 | 0.2 ± 0.4 | 0.0 ± 0.0 | 0.1 ± 0.2 | 0.0 ± 0.0 | 0.4 ± 1.1 |
| HPDs duration [s] | 4.3 ± 5.4 | 2.4 ± 5.1 | 0.6 ± 0.8 | 10.6 ± 17.3 | 0.3 ± 0.4 | 10.5 ± 26.6 |
| low-HPDs | **week 1** | **week 2** | **week 3** | **week 4** | **week 5** | **2 months** |
| Spike trains number [n] | 10.7 ± 7.2 | 12.0 ± 9.9 | 7.1 ± 6.2 | 7.4 ± 6.8 | 6.4 ± 4.3 | 7.2 ± 5.7 |
| Spike trains duration [s] | 20.4 ± 14.1 | 22.5 ± 20.6 | 12.5 ± 12.7 | 17.1 ± 23.4 | 11.1 ± 9.5 | 12.6 ± 10.6 |
| HPDs number [n] | 0.1 ± 0.3 | 0.2 ± 0.3 | 0.1 ± 0.2 | 0.1 ± 0.2 | 0.1 ± 0.2 | 0.1 ± 0.1 |
| HPDs duration [s] | 2.0 ± 3.8 | 2.8 ± 3.5 | 7.0 ± 20.4 | 2.3 ± 2.4 | 2.2 ± 5.6 | 1.2 ± 1.7 |

***Supplemental Table 3. Analysis of the cortical electrode in female mice after IHKA*** ***presented as mean ± standard deviation.***
